# Examination of collegiate student-athlete concussion reporting intentions and behavior

**Published:** 2020-04-16

**Authors:** Michelle L. Weber Rawlins, David Welch Suggs, Laura Bierema, L. Stephen Miller, Fred Reifsteck, Julianne D. Schmidt

**Affiliations:** ^1^A.T. Still University, Mesa, AZ; ^2^Grady College of Journalism and Mass Communication, Grady Sports Media Initiative, University of Georgia, Athens, Georgia, United States; ^3^Program Adult Learning, Leadership, and Organization Development, University of Georgia, Athens, Georgia, United States; ^4^Department of Psychology, University of Georgia, Athens, Georgia, United States; ^5^University Health Center, University of Georgia, Athens, Georgia, United States; ^6^UGA Concussion Reserach Laboratory, Department of Kinesiology, University of Georgia, Athens, Georgia, United States

**Keywords:** reporting behaviour, reporting intention, sport-related concussion, theory of planned behavior

## Abstract

**Background:**

Clinicians rely on student-athletes to self-report concussion symptoms, but more than 50% of concussions go undisclosed.

**Aim:**

The aim of this study was to determine whether knowledge, attitudes, subjective norms, self-efficacy, social identity, and athletic identity explain variability in student-athlete concussion reporting intentions and behavior.

**Materials and Methods:**

One hundred and forty-seven Division I and II collegiate student-athletes (male=23, female=56, missing=168; age=19.04±1.98 years) completed survey segments regarding the following predictor variables: Concussion knowledge, attitudes, subjective norms, self-efficacy, social identity, and athletic identity; and the following criterion variables: Reporting intentions (symptom and concussion reporting) and reporting behavior (symptom and concussion reporting) (completion rate=29.2%). Separate linear and logistic regressions were performed for each criterion variable. Backward elimination Akaike Information Criterion was applied to determine the best fit model.

**Results:**

A one-point increase in knowledge, attitudes, and self-efficacy predicted a significant 0.55, 0.23, and 0.31 increase in symptom reporting intentions, and 0.24, 0.30, and 0.33 increase in concussion reporting intentions of concussion reporting. As self-efficacy increased, symptom reporting behavior increased by 140%. When knowledge increased, concussion reporting behavior decreased by 23%. Whereas when subjective norms increased, concussion reporting behavior increased by 23%.

**Conclusions:**

A student-athletes’ confidence, or self-efficacy, was a frequent predictor of concussion reporting intentions and behavior.

**Relevance for Patients:**

Clinicians should aim to increase student-athlete knowledge, attitudes, and subjective norms, but most importantly their confidence in reporting concussions.

## 1. Introduction

Sport-related concussions diagnosis is often challenging as individuals with concussion experience a range of signs and symptoms [1]. The presence of those symptoms is easily identified in some cases (e.g., stumbling, and confusion about the next play), allowing for immediate recognition and diagnosis. However, for less visible or physical symptoms, such as headache or “feeling in a fog,” clinicians must rely on student-athletes to self-report symptoms. Previous studies have suggested that among student-athletes approximately 50% of concussions go unreported [2-8].

The Theory of Planned Behavior has been used as a framework to describe concussion reporting behavior [9-14]. According to the theory, behavior is best predicted by a person’s intention to perform that behavior, which is shaped by three factors: (1) Attitudes (e.g., the student-athlete’s belief regarding what will happen if they report a concussion), (2) subjective norms (e.g., the student-athlete’s belief regarding what others expect him/her to do), and (3) self-efficacy (e.g., the student-athlete’s belief regarding his/her ability to report a concussion) [15]. The previous concussion education efforts have focused primarily on improving student-athletes’ concussion knowledge, but several studies have found that knowledge has little influence on intention to report concussion [11,14,16]. Student-athletes often fail to report concussions because they believe the injury is not serious, fear missing games, fail to recognize concussion symptoms, and fear letting down teammates and coaches [2,14,17]. These beliefs support the hypothesis that attitudes, subjective norms, and self-efficacy are factors affecting the decision to report a concussion [9,14,18].

Social identity theory may also help explain the decision-making process in health behavior [19,20]. Individuals partly derive their identity from their place in society and desire positive social identity [21] Student-athletes with strong athletic identity may view injury, specifically concussion, as a threat to their athletic status, thus decreasing their likelihood of reporting a potential concussion [22].

The primary aim of this study was to determine the extent to which student-athlete knowledge, attitudes, subjective norms, self-efficacy, social identity, and athletic identity predict intentions and behavior regarding concussion reporting. We hypothesized that these factors would significantly predict concussion reporting intentions and behavior.

## 2. Materials and Methods

Eight hundred and forty-six varsity student-athletes at a Division I and Division II universities in the southeast were invited to complete a survey. E-mail addresses were obtained from the athletic departments. Institutional Review Board approval was obtained and participants consented before starting the survey. The survey was created, distributed, and maintained through Qualtrics Survey Software (Qualtrics Lab, Inc., Provo, UT). Weekly reminder emails were sent for 1 month. To address an initially low response rate, Division I student-athletes who did not participate through email were approached again for survey participation during their annual concussion baseline assessment between April 2016 and February 2017. Division II student-athletes received a paper survey to complete in fall 2016. When administered during concussion baseline testing, researchers verbally asked for interest in survey participation. If student-athletes agreed, the researcher opened the Qualtrics survey through web browser and the student-athlete completed the survey alone in a quiet room. Paper surveys were circulated through the athletic trainer at the Division II site. The previous studies suggest that paper and electronic surveys elicit similar responses [23-26].

The survey was divided into eight sections: Knowledge, attitudes, subjective norms, self-efficacy, social identity, athletic identity, intentions (symptom and concussion reporting), and behavior (symptom and concussion reporting). All survey items are listed in Table 1. Symptom reporting measures include listing common concussion symptoms and asking participants if they intended to report or had reported a concussion. Concussion reporting measures included asking student-athletes directly if they intended to or actually reported a concussion or “bell-ringer/ding.” Presentation of item blocks regarding each variable and items within blocks (where order did not matter) were randomized. Knowledge, attitudes, subjective norms, self-efficacy, athletic identity, and intention sections were rated on a seven-point Likert-scale (1=“strongly disagree” and 7=“strongly agree”). Social identity and behavior measure choices are described below. A pilot administration of the survey was given to 64 student-athletes at the Division I university. To determine test-retest reliability, a subsample of 12 participants completed the survey twice, 2 weeks apart. The survey had fair to excellent item level internal consistency and test- and re-test reliability for knowledge (Cronbach α=0.64, ICC_2,1_:0.62), attitudes (α=0.73, ICC_2,1_:0.62), subjective norms (α=0.82, ICC_2,1_:0.93), self-efficacy (α=0.95, ICC_2,1_:0.73), social identity (α=0.74, ICC_2,1_:0.85), intentions (α=0.92, ICC_2,1_:0.52), and behavior (α=0.88, ICC_2,1_:0.45).

**Table 1:** Mind matters challenge survey tool.

Concussion knowledge [14,18,27]		

Directions: These questions contain statements about concussions that may or may not be true. Please rate how strongly you agree with each statement.		
1	People who have had a concussion are more likely to have another concussion.	Strongly disagree (1) - Strongly agree (7)
2	There is a possible risk of death if a second concussion occurs before the first one has healed.	
3	A concussion cannot cause brain damage unless the person has been knocked out.	
4	The brain never fully heals after a concussion.	
5	It is easy to tell if a person has a concussion by the way the person looks or acts.	
6	Symptoms of a concussion can last for several weeks.	
7	Resting your brain by avoiding things such as playing video games, texting, and doing schoolwork is important for concussion recovery.	
8	After a concussion occurs, brain imaging (e.g., computer assisted tomography scan, magnetic resonance imaging, and X-ray) typically shows visible physical damage to the brain (e.g., bruise, and blood clot).	
9	A concussion may cause an athlete to feel depressed or sad.	
10	Once an athlete feels &“back to normal,&” the recovery process is complete.	
11	Even if a player is experiencing the effects of a concussion, performance on the field of play will be the same as it would be had the player not experienced a concussion.	
12	Concussions pose a risk to an athlete&’s long-term health and well-being.	
13	A concussion can only occur if there is a direct hit to the head.	

**Attitudes [9,14,18]**		

**Directions: Please rate how strongly you agree with each statement.**		

1	If I report what I suspect might be a concussion, I will hurt my team&’s performance.	Strongly disagree (1) - Strongly agree (7)
2	If I report what I suspect might be a concussion, I will not be allowed to start playing or practicing when I think I&’m ready.	
3	If I report what I suspect might be a concussion, I will lose my spot in the line-up.	
4	If I report what I suspect might be a concussion, my teammates will think less of me.	
5	The sooner I report a concussion the sooner I&’ll be back at full strength.	
6	If I report what I suspect might be a concussion, I will be held out of upcoming games even if it is NOT a concussion.	
7	If I report what I suspect might be a concussion, my teammates will think I made the right decision.	
8	If I report what I suspect might be a concussion, I will be better off in the long run.	

**Subjective norms [14,18,27]**		

**Directions: Please read each of the following scenarios and rate how strongly you agree or disagree with the statements that follow.**		

**Scenario 1: Athlete M experienced a concussion during the first game of the season. Athlete O experienced a concussion of the same severity during the semifinal playoff game. Both athletes had persisting symptoms.**		Strongly disagree (1) - Strongly agree (7)

1	My teammates would feel that Athlete M should have returned to play during the first game of the season.	
2	Most athletes would feel that Athlete M should have returned to playing during the first game of the season.	
3	My teammates would feel that Athlete O should have returned to play during the semifinal playoff game.	
4	Most athletes would feel that Athlete O should have returned to playing during the semifinal playoff game.	

**Scenario 2: Player R experiences a concussion during a game. Coach A decides to keep Player R out of the game. Player R&’s team loses the game.**		

5	My teammates would feel that Coach A made the right decision to keep Player R out of the game.	
6	Most athletes would feel that Coach A made the right decision to keep Player R out of the game.	

**Scenario 3: Athlete R experiences a concussion. Athlete R&’s team has an athletic trainer on the staff.**		

7	My teammates would feel that the athletic trainer, rather than Athlete R, should make the decision about returning Athlete R to play.	
8	Most athletes would feel that the athletic trainer, rather than Athlete R, should make the decision about returning Athlete R to play.	

**Athlete H experienced a concussion and has a game later in the day. He is still experiencing symptoms of concussion. However, Athlete H knows that if he tells his coach about the symptoms, his coach will keep him out of the game.**		

9	My teammates would feel that Athlete H should tell his coach about the symptoms.	
10	Most athletes would feel that Athlete H should tell his coach about the symptoms.	

**Scenario 4: Athlete H experienced a concussion and has a game later in the day. He is still experiencing symptoms of concussion. However, Athlete H knows that if he tells his coach about the symptoms, his coach will keep him out of the game.**		

11	My teammates would feel that Athlete H should tell his coach about the symptoms.	
12	Most athletes would feel that Athlete H should tell his coach about the symptoms.	
13	My teammates would continue playing while also having a headache that resulted from a minor concussion.	
14	Most athletes would continue playing while also having a headache that resulted from a minor concussion.	

**Self-efficacy [14,18]**		

**Directions: Please rate how strongly you agree with each statement.**		

1	I am confident in my ability to recognize when I have symptoms of a concussion.	Strongly disagree (1) - Strongly agree (7)
2	I am confident in my ability to report symptoms of a concussion, even when I really want to keep playing.	
3	I am confident in my ability to report symptoms of a concussion, even when I think my teammates want me to play.	
4	I am confident in my ability to report symptoms of a concussion, even if I do not think they are all that bad.	
5	I am confident in my ability to report specific symptoms, even if I am not sure that it is actually a concussion.

**Athletic identity [33]**		

**Directions: Please rate how strongly you agree with each statement.**		

1	I consider myself an athlete.	Strongly disagree (1) - Strongly agree (7)
2	I have many goals related to sport.	
3	Most of my friends are athletes.	
4	Sport is the most important part of my life.	
5	I spend more time thinking about sport than anything else.	
6	I need to participate in sport to feel good about myself.	
7	Other people see me mainly as an athlete.	
8	I feel bad about myself when I do poorly in sport.	
9	Sport is the only important thing in my life.	
10	I would be very depressed if I were injured and could not compete in sport.

**Social identity [29-32]**		

**Please indicate your willingness to engage in the following activities with a recently concussed athlete if given the opportunity:**		

1	Compete with a recently concussed athlete as a teammate (e.g., be in the starting lineup together).	Not at all willing (1) - Extremely willing (7)
2	Have a recently concussed athlete on your team.	
3	Confide in a recently concussed athlete.	
4	Be in a study group with a recently concussed athlete.	
5	Have a recently concussed athlete visit your home/apartment/residence hall.	
6	Visit a recently concussed athlete&’s home/apartment/residence hall.	
7	Have a recently concussed athlete as a team captain.	
8	Attend a forum for athletes suffering from concussion.

**Symptom reporting intentions [9,14,18]**		

**Directions: Please rate how strongly you agree with the following statement: &“I would stop playing and report my symptoms if I sustained an impact that caused me to.&”**		

1	See stars.	Strongly Disagree (1) - Strongly Agree (7)
2	Vomit or feel nauseous.	
3	Have a hard time remembering things.	
4	Have problems concentrating on the task at hand.	
5	Feel sensitive to light or noise.	
6	Have a headache.	
7	Experience dizziness or balance problems.	
8	Feel sleepy or in a fog.	

**Concussion reporting intentions [9,14,18]**		

**When I experience possible concussion symptoms&…**		

1	I intend to report.	
2	I plan to report.	
3	I will make an effort to report.	

**Symptom reporting behavior [9,14,18]**		

**Directions: Please read the following statements. Please circle YES if the following has occurred to you IN THE PAST 365 DAYS and circle NO if it has not occurred to you THIS SEASON.**		
1	Dizziness after an impact.	Yes or No
2	Had my bell rung.	
3	Lost consciousness or blacked out after an impact.	
4	Saw stars after an impact.	
5	Vomited or felt nauseous after an impact.	
6	Forgot what to do in the rink after an impact.	
7	Had a headache at least once during the week after an impact.	
8	Had problems studying, concentrating or doing class work after an impact.	
9	Experienced any of these symptoms after an impact but did not immediately tell a coach or athletic trainer (e.g., kept playing in a practice or game).	
10	Continued to experience any of these symptoms the day after a hit but did not tell a coach or athletic trainer.

**Concussion reporting behavior [9,14,18]**		

11	In the past 365 days, how many concussions do you think you have experienced? _____	Fill in the blank
12	In the past 365 days, how many of the possible concussions you experienced did you report to a medical professional (doctor, athletic trainer, etc.) or a coach? _____	
13	In the past 365 days, how many times have you had your &“bell rung&” or been &“dinged?&” ______	
14	In the past 365 days, how many of the &“bell rung&” or &“dinged&” episodes you experienced did you report to a medical professional (doctor, athletic trainer, etc.) or a coach? ______	
15	Reason for not reporting a concussion, &“bell rung&” episode, or &“dinged&” episode:	
	____ Did not think it was serious	
	____ Did not know it was a concussion	
	____ Did not want to be pulled out of the game/practice	
	____ Did not want to be pulled from future games/practices	
	____ Did not want to let your teammates down	
	____ Would have if it as less important game/practice	
	____ Other? If so why? ___________________________	

### 2.1. Knowledge measure

This section was obtained from previously published research and contained 13 items [14,18,27]. Sample question included “A concussion may cause an athlete to feel depressed or sad.” Two questions were not included in concussion knowledge score: “The brain never fully heals after a concussion,” and “Concussions pose a risk to an athlete’s long-term health and well-being.” Responses had high variability and scientific literature and medical consensus do not yet fully support a “correct” response [1,28].

### 2.2. Attitudes measure

This section included eight items and was obtained from previous studies [9,14,18]. Participants were asked to rate their agreement to items such as “If I report what I suspect might be a concussion, I will hurt my team’s performance.”

### 2.3. Subjective norms measure

This section included four scenarios and 12 items [14,18,27]. Based on the scenario, student-athletes rated agreement with how “my teammates” or “most athletes” may act given the scenario. For example, student-athletes answered how strongly they agreed with the statement “My teammates would feel that the athletic trainer, rather than Athlete R, should make the decision about returning Athlete R to play.”

### 2.4. Self-efficacy measure

This section included five items utilized from the previous studies [14,18]. Participants were asked to rate agreement for items such as “I am confident in my ability to recognize when I have symptoms of a concussion.”

### 2.5. Social identity measure

Social identity was assessed using eight items [29-32] and included statements regarding the student-athletes’ willingness to “Visit a recently concussed athlete’s home/apartment/residence hall.” Items were based on a seven-point Likert scale (1=“not willing” and 7=“extremely willing”).

### 2.6. Athletic identity measure

Athletic identity was measured with ten items [33]. Student-athletes rated their agreement for an item such as “I consider myself an athlete.”

### 2.7. Reporting intentions measure

This measure was divided into symptom and concussion reporting sections, and used from Kroshus *et al*. [14,18] and Register-Mihalik *et al*. [9]. The symptom reporting intentions section included eight items rated on level of agreeance to the following statement “I would stop playing and report my symptoms if I sustained an impact that would cause me to…,” for example, “See stars,” or “vomit or feel nauseous.” The concussion reporting intention section included three items of “I intend to report,” “I plan to report,” and “I will make an effort to report.”

### 2.8. Reporting behavior measure

This measure was also divided into symptom and concussion reporting sections [9,14,18]. The symptom reporting behavior section included ten items with “yes” or “no” answer and a general prompt: “Please circle yes if the following has occurred to you within the past 365 days and circle no if it has not occurred to you within the past 365 days.” Example items include “Dizziness after an impact” and “Had my bell rung.” The last two items asked participants to select “yes” or “no” if they “Experienced any of these symptoms after an impact but did not immediately tell a coach or athletic trainer” or “Continued to experience any of these symptoms the day after a hit but did not tell a coach or athletic trainer.” Concussion reporting behavior items included open-ended questions such as “How many concussions do you think you have experienced?” and “How many of the possible concussions you experienced did you report to a medical professional (doctor, athletic trainer, etc.) or a coach?” The same questions regarding number of dings/”bell-ringers” experienced and how many dings/”bell-ringers” reported was also asked.

### 2.9. Data analysis

Data analysis was conducted using RStudio, Inc. (v 3.3.3, Murray Hills, NJ). Knowledge, attitude, subjective norms, self-efficacy, social identify, athletic identity, symptom reporting intention, and concussion reporting intention item responses were averaged separately across items (minimum of one and maximum of seven for each). Items were reverse coded when necessary. In the event participants skipped questions for seven-point Likert-scale items, the neutral number (four) replaced the missing value (<1% replaced). Based on findings by Bennet *et al*. [34], Poh *et al*. [35], and Yates [36], after statistical consultation, and since the neutral values were near the medians, we believed that the Fisher-Yates method replacing missing values with the neutral number was appropriate.

For symptom reporting behavior, student-athletes that reported experiencing concussion related symptoms in the past 365 days (items one through eight) were categorized as either “reporter” or “non-reporter” based on their symptom reporting (items nine and ten). Student-athletes that reported that they had not experienced concussion related symptoms in the past 365 days were labeled as “no event” and were excluded from analysis for behavior only, since they did not have an event to report or conceal. Concussion reporting behavior was calculated the ratio between the total number of concussions, dings, and bell-ringers reported divided by number of concussions, dings, and bell-ringers experienced.

### 2.10. Statistical analysis

Descriptive statistics for age, gender, sport, and concussion reporting behavior were calculated. Two separate multivariate linear regressions were conducted with all eight variables predicting symptom and then concussion reporting intentions. Two separate logistic regressions were conducted with the eight variables predicting symptom and then concussion reporting behavior. Due to over-parametrization, backward elimination Akaike Information Criterion (AIC) was applied to each model. AIC is an estimator of the goodness of fit or quality to account for model complexity. This procedure does not use p-value in selection criteria, so a predictor variable with *P*<0.05 may enter the final model as an influential factor [37]. “Average” student-athlete results were calculated by inserting mean values for predictor variables into the regression equation.

## 3. Results

In this study, 343 of 846 student-athletes accessed the survey. Ninety-six student-athletes were excluded from analysis because too many variable fields were missing (i.e., missing more than one survey section). The remaining 247 student-athletes were included in analysis for a completion rate of 29.20% (n=247/846), 119 from a Division I site and 128 student-athletes from the Division II site. Table 2 includes additional demographics results. Table 3 includes descriptive results for each predictor variable.

**Table 2:** Participant demographics for separated by division.

Demographic	Division I (n=119)	Division II (n=128)
Age	19±2.0 years (n=69, missing=50)	20±1.53 years (n=3, missing=125)
Gender		
Males	18.5% (n=22)	0.8% (n=1)
Females	45.4% (n=54)	1.6% (n=2)
Missing	36.1% (n=43)	97.7% (n=125)
Sport		
Baseball	8.4% (n=10)	20.3% (n=26)
Equestrian	6.7% (n=8)	--
Football	8.4% (n=10)	48.4% (n=62)
Golf	6.7% (n=8)	0% (n=0)
Gymnastics	3.4% (n=4)	--
Soccer	12.6% (n=15)	2.3% (n=3)
Softball	7.6% (n=9)	2.3% (n=3)
Swimming and dive	10.9% (n=13)	--
Tennis	2.5% (n=3)	0% (n=0)
Track and field/Cross country	25.2% (n=30)	5.5% (n=7)
Volleyball	7.6% (n=9)	9.4% (n=12)
Missing	0% (n=0)	11.7% (n=15)

--:Not a sport at this site

**Table 3:** Descriptive results for predictor and criterion variables.

Variable	Mean&±standard deviation	Median	Nearest response category to median	95&% confidence interval
Predictor variables				
Knowledge	4.99&±0.63	5.00	Somewhat agree	4.91-5.07
Attitudes	4.94&±0.81	4.88	Somewhat agree	4.84-5.04
Subjective norms	5.03&±0.86	5.08	Somewhat agree	4.92-5.14
Self-efficacy	5.44&±1.12	5.60	Agree	5.30-5.58
Social identity	4.60&±1.36	4.63	Somewhat willing	4.43-4.77
Athletic identity	5.01&±0.92	5.10	Somewhat agree	4.90-5.12
Criterion variables				
Intentions				
Symptom reporting	5.21&±1.42	5.63	Agree	5.03-5.39
Concussion reporting	6.01&±1.00	6.00	Agree	5.88-6.13
Behavior				
Concussion reporting	0.45&±0.42	0.36	NA	0.29-0.62

The neutral number (four) replaced missing values which accounted for less than 1&%.

### 3.1. Reporting intentions

**Figure 1:**
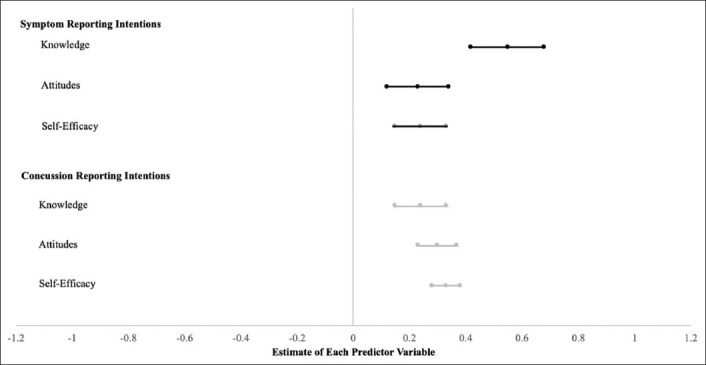
Forest plot with regression results of predictor estimates with confidence intervals for reporting intentions.

Results based on AIC analysis are presented in Table 4. Figure 1 includes a forest plot with significant regression results of predictor estimates with confidence intervals for reporting intentions. Knowledge, attitudes, and self-efficacy significantly predicted symptom reporting intentions. For every one-point increase in knowledge, attitudes, and self-efficacy, a respective 0.55, 0.23, and 0.31 increase in symptom reporting intentions is expected. On average, student-athletes had a symptom reporting intention of 5.21 (“somewhat agree”). Similarly, knowledge, attitudes, and self-efficacy also significantly predicted concussion reporting intentions. For every one-point increase in knowledge, attitudes, and self-efficacy, concussion reporting intention would be expected to increase by 0.24, 0.30, and 0.33, respectively. On average, student-athletes had concussion reporting intentions scores of 6.01 (“agree”). Social and athletic identity were not significant predictors of symptom or concussion reporting intentions.

**Table 4:** Significant predictors of criterion variables based on Akaike Information Criteria (AIC).

Criterion variable	Significant predictors based on AIC	Estimate	Standard error	*t*-value	*P*-value
Intentions					
Symptom reporting	Intercept	−0.38	0.78	−0.48	*P*=0.63
	Knowledge	0.55	0.13	4.12	*P*<0.001
	Attitudes	0.23	0.11	2.19	*P*=0.029
	Self-efficacy	0.31	0.08	4.10	*P*<0.001
Concussion reporting	Intercept	1.55	0.51	3.02	*P*=0.003
	Knowledge	0.24	0.09	2.78	*P*=0.006
	Attitudes	0.30	0.07	4.26	*P*<0.001
	Self-efficacy	0.33	0.05	6.58	*P*<0.001
Behavior				Exponential estimate	
Symptom reporting	Intercept	−4.04	1.76		*P*=0.02
	Self-efficacy	0.87	0.33	2.40	*P*=0.009
Concussion reporting	Intercept	−0.04	1.05		*P*=0.968
	Knowledge	−0.27	0.18	0.77	*P*=0.128
	Subjective norms	0.21	0.14	1.24	*P*=0.140

The neutral number (four) replaced missing values which accounted for &<1&%

### 3.2. Reporting behavior

Nearly 75% of concussive events (74.6%, n=94/126 events) that occurred within 365 days before survey completion were reported. However, only 26% of “dings/bell-ringers” within 365 days of being surveyed were reported (25.8%, n=70/271 events). When combined, approximately 41.3% of concussions, dings, and ‘‘bell-ringers” were reported (n=164/397 events). One hundred and nine participants experienced a concussion, ding/”bell-ringer,” or both. The most frequent reason for not reporting a concussion was “did not think it was serious” (61.5%, n=67/109), followed by “did not know it was a concussion” (30.3%, n=33/109), “did not want to be pulled out of the game/practice” (29.4%, n=32/109), “did not want to be pulled from future games/practices” (25.7%, n=28/109), “did not want to let teammates down” (18.3%, n=20/109), “would have if it was less important game/practice” (9.2%, n=10/109), and “other” (5.5%, n=6/109).

**Figure 2:**
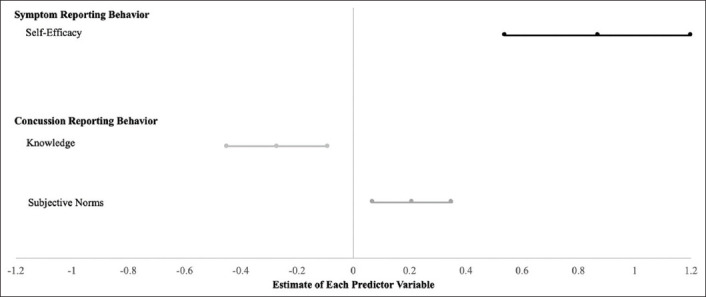
Forest plot with regression results of predictor estimates with confidence intervals for reporting behavior.

In the best fit model from logistic analysis with AIC, self-efficacy was a significant predictor of symptom reporting behavior (Table 4). Figure 2 includes a forest plot with significant regression results of predictor estimates with confidence intervals for reporting behavior. Regardless of any predictor variables, 62% (n=33/53) of student-athletes were “reporters.” Exponential estimates indicated that every one-point increase in self-efficacy increases, the odds of being a “reporter” increased by 140%. Knowledge and subjective norms were the most influential predictors of concussion reporting behaviors. Contrasting with our previous finding regarding symptom and concussion reporting intentions, a one-point increase in knowledge reduced the odds of reporting a concussion by 23%. On the other hand, if subjective norms increased by one point, the odds of reporting a concussion increased by 24%. Social and athletic identity was not significant predictors of symptom or concussion reporting behavior.

## 4. Discussion

Our study identified factors that appear to influence concussion reporting intentions and behavior among collegiate student-athletes. Student-athletes’ self-efficacy significantly influenced reporting intentions and behavior. Self-efficacy is defined as “the perceived ease or difficulty of performing the behavior…”[15] (p. 188) Although to a lesser extent, knowledge and attitudes also influenced concussion reporting intentions. This study adds to the body of literature in that it is novel to examine concussion reporting intentions and behavior in a sport diverse population, and examine how knowledge, attitudes, subjective norms, self-efficacy, social identity, and athletic identity explain variability in student-athlete concussion reporting intentions and behavior.

A student-athletes’ belief they can perform the behavior, or self-efficacy, was a frequent predictor of concussion reporting intentions and behavior. This has clinical importance in that clinicians, including athletic trainers, should aim to increase student-athlete knowledge, attitudes, and subjective norms, but most importantly their belief in carrying out actual concussion reporting due to our findings. Many concussion educational tools exist, but few currently aim to address variables other than knowledge. Educational materials should include knowledge components, such as signs and symptoms of a concussion, common health-care providers whom their injury could be reported to, and that dings/”bell-ringers” may be concussions. To report a concussion, student-athletes must first know what a concussion is and how to report it [8,10,14]. Although the previous research found that knowledge having little influence on concussion reporting behavior [11,14,16], our results indicated knowledge was a fairly influential predictor. These results, and that of Register-Mihalik *et al*. [8], indicate athletes that possess base knowledge have stronger intentions to report their concussion. Perhaps increasing knowledge may increase the understanding of the seriousness and importance of reporting the injury. In the case of concussion reporting behavior, there was a negative association with knowledge – that is, the higher knowledge, the fewer concussions reported. This finding further highlights that knowledge does not necessarily translate to reporting a concussion. Knowledge may not be translating to behavior because many social factors may also influence concussion reporting.

Self-efficacy was the most frequent and strongest predictor of concussion reporting intentions and behavior, echoing the findings of Register-Mihalik *et al*. [9]. Intervening to improve student-athletes’ confidence that reporting a concussion will address their symptoms and help them return to play may be a useful strategy for improving concussion reporting. If student-athletes are confident that their injury is a concussion, are sure of steps required once a concussion has been identified, and that reporting is required of them, this may increase their action of reporting. The consistency across models and the effect size of self-efficacy in our study underscores how important confidence in reporting is and suggests that concussion education efforts should strive to improve this confidence in seeking care. To increase self-efficacy or confidence in reporting, educational materials could state “you know what to do,” or have an interactive section. In this interactive section, student-athletes could choose appropriate actions given a scenario. Immediate feedback could be given if they choose to report a concussion such as “Great job. You are correct in that this is a concussion and should be reported” or “you did the right thing.” If a student-athlete chooses to conceal the injury given the scenario, immediate feedback indicating the scenario injury is likely a concussion, and next steps can be taken.

Attitudes and subjective norms were the second most common factor to predict intentions and behavior, but the effects were mostly small. Attitudes relate to one’s positive or negative views regarding an action and subjective norms are the person’s belief regarding what is expected of them to do from others [15]. Similar to our results, Register-Mihaik *et al*. [8,9] found that favorable attitudes toward concussions were associated with an intention to report a concussion. These results indicate if student-athletes obtain more positive views of concussions and understand subjective norms, they may be more influenced to report their injury. In addition, educational materials could contain statements about what the larger body of student-athletes expects of them regarding concussion reporting. Statements could include having student-athletes say “I want my fellow teammates to report their injury” or “you will be better off in the long run if you report your concussion.”

In our sample, only 41% of concussion, dings, and bell ringers were reported to a medical professional or coach. It has been estimated that as many as 50% of concussions go unreported in student-athletes [2-8]. When analyzed separately, we found that 75% of concussions were reported, but only 26% of dings/”bell-ringers” were reported. These results indicate that confusion still exists regarding what a concussion is and reveals a misunderstanding among student-athletes that indicate that they do not view dings/”bell-ringers” as concussions [38]. Even though describing concussions dings or “bell-ringers” is seen as minimizing the severity of the injury and should be avoided by professional [28], when asking student-athletes if they have ever had a ding/”bell-ringer” or if they are experiencing symptoms of a dings/”bell-ringers” may be useful for gathering a full background of head injury.

Student-athletes with higher athletic identity have been found to under-report concussion symptoms [22], which contrast our results. Neither social nor athletic identity was significant predictors of intentions or behavior regarding concussion reporting. Our findings may have yielded different results due to sample. Our sample included collegiate student-athletes of all sports, whereas Kroshus, Kubzansky, Goldman, and Austin [22] examined Division I men’s ice hockey student-athletes. Student-athletes from our sample were “somewhat willing” to interact with concussed student-athletes. Clinicians should focus on improving concussion reporting culture in individual student-athletes themselves, but also team wide to ensure support for concussed student-athletes from their teammates.

### 4.1. Future directions

Future research should focus on implementation of self-efficacy into concussion education by designing programs aimed at increasing student-athletes’ confidence in concussion reporting. Second, this study only accounts for variables of knowledge, attitudes, subjective norms, self-efficacy, social identity, and athletic identity. Additional research should be performed on other variables that may influence concussion reporting behavior such as perceived severity of health consequences following a concussion or media influence.

### 4.2. Limitations

Although data from this study included one of the largest total samples to date and from the widest variety of collegiate student-athletes, data from student-athletes are only from two sites in a single geographical location. Student-athletes at these two sites may not accurately reflect knowledge, attitudes, subjective norms, self-efficacy, social identity, and athletic identity, intentions and behavior of all or the majority collegiate student-athletes. Second, our demographic results include missing results due to error and future research should include full demographic data and include a diverse sample. Furthermore, our sample included Divisions I and II sites. Future research should aim to examine knowledge, attitudes, subjective norms, self-efficacy, social identity, and athletic identity, intentions and behavior in Division III student-athletes as well.

## 5. Conclusion

Authors estimate approximately 50% of concussions goes unreported [2-8]. In our sample, 41% of concussions (concussions and bell-ringers/dings combined) were reported. Self-efficacy, or confidence in reporting, along with knowledge, attitudes, and subjective norms play a large role in student-athletes’ intention and behavior to report a concussion. Clinicians, including athletic trainers, can use this information to increase a student-athletes’ confidence in concussion reporting. Future actions should include designing concussion educational interventions to increase self-efficacy regarding concussion reporting.

### Funding Source

This project was conducted with support from the National Collegiate Athletic Association-Department of Defense Research Grand Challenge: Changing Attitudes about Concussions in Young and Emerging Adults.

### Conflicts of Interest

Michelle Weber Rawlins has received stipend and travel funds from The National Collegiate Athletic Association-Department of Defense Research Grand Challenge: Changing Attitudes about Concussions in Young and Emerging Adults Grant. Julianne Schmidt was the principal investigator in receiving this grant, and too received travel funds. The grant was the research funding source for the associated manuscript being submitted. David Welch Suggs also received travel funding from this grant. Laura Bierema, L. Stephen Miller, and Fred Reifsteck have no conflicts of interest to declare.
